# Development and first validation of the Portuguese version of the Big Three Perfectionism Scale–Short Form (BTPS-SF)

**DOI:** 10.1192/j.eurpsy.2023.420

**Published:** 2023-07-19

**Authors:** A. T. Pereira, M. J. Brito, C. Marques, A. I. Araujo, C. Cabaços, M. Carneiro, A. P. Amaral, A. Macedo

**Affiliations:** ^1^ Institute of Psychological Medicine, Faculty of Medicine, Coimbra University; ^2^ Coimbra Institute for Biomedical Imaging and Translational Research; ^3^Coimbra Health School, Polytechnic Institute of Coimbra, Coimbra, Portugal

## Abstract

**Introduction:**

The Portuguese version of the Big Three Perfectionism Scale (BTPS), a 45-item self-report measure of rigid, self-critical, and narcissistic perfectionism, presented good reliability, construct and concurrent validity both in a sample of university students (Lino, Pereira et al. 2018) and of adults from the general population (Oliveira, Pereira et al. 2021).

**Objectives:**

To develop and validate a Portuguese brief version of the BTPS, the Big Three Perfectionism Scale–Short Form (BTPS-SF) in a sample of university students.

**Methods:**

The procedure followed to select items for the short version was based on the 45-items BTPS confirmatory factorial analysis (Lino, Pereira et al. 2018). Following Feher et al. (2020) strategy, with Canadian university students, we retained between one and two from each of the 10 perfectionism facets in the BTPS, 16 items in total. The 16 items selected had loadings ranging from .63 to .88 (Lino, Pereira et al. 2018), thus meeting the suggested requirement of high loadings being above .60 in magnitude (Afifi et al. 2011).

Participants were 633 Portuguese students (medicine, dentistry and health technologies; 82.1% girls; mean age=21.25±3.115); they answered an online survey including the BTPS and the Depression Anxiety and Stress Scale (DASS; Xavier et al. 2017).

**Results:**

Confirmatory Factor Analysis showed that both the first (χ2/df=3.074; RMSEA=.0573, p<.001; CFI=.9591; TLI=.9478, GFI=.9465) and the second order (χ2/df=3.714; RMSEA=.0655, p<.001; CFI=.9482; TLI=.9317, GFI=.9318) models presented good fit indexes. The Cronbach’s alfas were: a=.865 for the total and .855, .829 and .750, respectively for F1 (rigid perfectionism), F2 (self-critical perfectionism) and F3 (narcissistic perfectionism). Pearson coefficient correlations with DASS total score were significant (p<.01), positive and moderate for the total 16-items- BTPS (r=.375), F1 (r=.285), F2 (r=.465) and low for F3 (r=.177). Correlation coefficients with Depression, Anxiety and Stress sub-scales presented the same pattern and magnitude.

**Conclusions:**

Due to its good validity and reliability, the Portuguese BTPS–SF is an efficient and useful alternative to the 55-item version. When it is not necessary to measure the ten facets, the BTPS-SF has the advantages of conciseness, brevity and ease of filling.

**Disclosure of Interest:**

None Declared

